# Prognostic Roles of *BRAF*, *KIT*, *NRAS*, *IGF2R* and *SF3B1* Mutations in Mucosal Melanomas

**DOI:** 10.3390/cells10092216

**Published:** 2021-08-27

**Authors:** Joanna P. Wróblewska, Dora Dias-Santagata, Adam Ustaszewski, Cheng-Lin Wu, Masakazu Fujimoto, M. Angelica Selim, Wojciech Biernat, Janusz Ryś, Andrzej Marszalek, Mai P. Hoang

**Affiliations:** 1 Department of Pathology, Poznan University Medical Sciences and Greater Poland Cancer Center, 61-701 Poznan, Poland; Joanna.wroblewska@wco.pl (J.P.W.); amars@ump.edu.pl (A.M.); 2 Department of Pathology, Massachusetts General Hospital and Harvard Medical School, Boston, MA 02114, USA; ddiassantagata@mgh.harvard.edu; 3 Institute of Human Genetics, Polish Academy of Sciences, 60-479 Poznan, Poland; adam.ustaszewski@igcz.poznan.pl; 4 Department of Pathology, National Cheng Kung University Hospital, College of Medicine, National Cheng Kung University, Tainan 70403, Taiwan; wujl.towalkwithwings@gmail.com; 5 Department of Pathology, Kyoto University Hospital, Kyoto 606-8507, Japan; fujimasa@kuhp.kyoto-u.ac.jp; 6 Department of Pathology, Duke University Medical Center, Durham, NC 27710, USA; angelica.selim@duke.edu; 7 Department of Pathology, Medical University of Gdansk, 80-210 Gdansk, Poland; biernat@gumed.edu.pl; 8 Department of Pathology, Maria Sklodowska-Curie National Research Institute of Oncology, 31-115 Cracow Branch, Poland; z5rys@cyf-kr.edu.pl

**Keywords:** mucosal melanoma, vulvovaginal, sinonasal, anorectal, *NRAS*, *KIT*, *BRAF*, *SF3B1*, *IGF2R*

## Abstract

Background: The prognostic value of commonly recurrent mutations remains unclear in mucosal melanomas. Methods: Clinicopathologic parameters of 214 cases of mucosal melanomas diagnosed in 1989–2020 in several clinical institutions were analyzed. *NRAS*, *KIT*, *BRAF*, *IGF2R* and *SF3B1* mutational analyses by Sanger sequencing and next generation sequencing-based assay were performed in a subset of cases. Results: Of the triple (*BRAF*, *NRAS*, *NF1*)-negative cases, *APC*, *KIT* and *KRAS* are detected mainly in sinonasal, vulvovaginal and anorectal melanomas, respectively. *NRAS*, *KIT*, *BRAF*, *IGF2R* and *SF3B1* mutations are detected in 19% (37/198), 22% (44/197), 12% (25/201), 16% (22/138) and 15% (20/133) of cases, respectively. In univariate analyses, advanced stage (*p* = 0.016), 65 years or older (*p* = 0.048) and presence of ulceration (*p* = 0.027) are significantly correlated with worse overall survival (OS), respectively. *NRAS* mutation significantly correlates with worse OS (*p* = 0.028) and worse melanoma-specific survival (MSS) (*p* = 0.03) for all cases of mucosal melanomas. In multivariate analyses, *NRAS* mutation remains as an independent predictor of worse OS (*p* = 0.036) and worse MSS (*p* = 0.024). Conclusion: *NRAS* mutation is a predictor of worse survival, independent of stage in mucosal melanomas. The significance of frequently mutated *IGF2R* in mucosal melanomas remains unclear.

## 1. Introduction

Mucosal melanomas encompass primary melanomas of the female genital tract (vulvar and vaginal melanoma), male genital tract (penile melanoma), head and neck region (sinonasal and oral melanoma), conjunctiva, upper gastrointestinal tract (esophagus, stomach, intestine), anorectal melanoma and urinary tract (urethra, urinary bladder) [1]. While previously considered to be a subtype of ocular melanoma, recent studies have shown that conjunctival melanomas have a similar tumorigenetic pathway as mucosal melanomas. Although mucosal melanomas are rare and constitute approximately 1.4% of all melanomas, the prognosis of patients with mucosal melanoma is poorer in comparison to cutaneous melanomas.

In line with prognosis, genetic alterations occurring in mucosal melanoma are different than those of cutaneous wild-type melanoma (*BRAF*, *NRAS* and *NF1* negative) [2,3]. The frequency of frequently mutated genes are as follows: *NRAS* (14–30%), *BRAF* (5–16%), *NF1* (16%), *KIT* (5–15%), *SF3B1* (12%), *TP53* (8.9%), *SPRED1* (7%), *ATRX* (6%) and *CHD8* (4%) [4,5,6,7,8,9,10]. Recently, *IGF2R* mutation was documented in 32% of 41 mucosal melanomas in comparison to 6% of 48 cutaneous melanomas [10].

In a series of 444 mucosal melanomas from a European population investigated by Sanger sequencing, *NRAS*, *KIT* and *BRAF* mutations were evenly distributed across the different mucosal melanoma subgroups [8]. The prognostic role of these commonly recurrent mutations in mucosal melanomas has only been studied in some series [6,7,11,12]. In a large series of 706 mucosal melanomas, *KIT* and *BRAF* mutational status did not correlate with overall survival (OS); however, *NRAS* was not analyzed in this series [6]. Correlation between *NRAS*, *BRAF* and *KIT* mutations and survival was not observed in prior series of sinonasal melanomas [7,12]. *KIT* mutation has been reported to be a marker of better progression-free survival in vulvar melanomas [11].

Although mutational status provides potential therapeutic targets, prognostic value of commonly recurrent mutations remains unclear in mucosal melanomas. In this study, we analyzed the prognostic role of *NRAS*, *KIT*, *BRAF*, *IGF2R* and *SF3B1* mutations in a series of mucosal melanomas.

## 2. Materials and Methods

The study was approved by Institutional Review Boards. Mucosal melanomas diagnosed between 1989 and 2020 were retrieved from the pathology archives of several clinical institutions in Japan, Poland, Spain, Taiwan and the United States. A total of 214 melanocytic tumors from 214 patients which were diagnosed with primary vulvar (73), vaginal (4), sinonasal (93), anorectal (31), conjunctival (8), urethral (1) and penile (4) melanomas were included in the study. Melanomas on the vulvar hair bearing skin are excluded. *NRAS*, *KIT* and *BRAF* mutational results of 72 sinonasal, 27 vulvar and 4 vaginal melanomas; and *SF3B1* results of 72 sinonasal melanomas from prior studies were included [11,12,13].

### 2.1. Clinical Findings and Histologic Features

The histopathologic diagnoses and following features were assessed by the contributing pathologists and confirmed by the corresponding author (MPH): ulceration, mitotic rate (per squared millimeters), lymphovascular invasion, and perineural invasion. The following data were extracted from medical records: age of the patients, lesion site, date of biopsy, disease status over time and at last follow-up (recurrence, metastasis) and any treatment.

### 2.2. Molecular Analyses

Sanger sequencing and next generation sequencing (NGS) were performed on subset of cases as outlined in Table 1. Deoxyribonucleic acid (DNA) was extracted from formalin-fixed paraffin-embedded tumors. For Sanger sequencing *NRAS* exons 1 and 2; *KIT* exons 9, 11, 13 and 17; *BRAF* exon 15; *SF3B1* codons 625 and 666 of exon 14, codon 700 of exon 15; and *IGF2R* exons 2, 6, 8, 16, 43 and 46 were amplified by polymerase chain reaction (PCR) with specific primers (Table S1) [12].

For *IGF2R* variant calling, raw genomic sequence data were obtained from NCBI (National Center for Biotechnology Information) Sequence Read Archive under Bioproject number PRJNA379027.10. The raw data consisted of 41 mucosal melanoma samples and were quality checked using FastQC software and mapped to the reference genome (GRCh38/hg38) using BWA-MEM [14]. Further analysis, including duplicated reads marking, base quality scores recalibration and eventually variant calling, was performed according to GATK best practices pipeline [15]. The preprocessed cohorts of variants were filtered using SnpSift [16]. Only detected variants with a high score for predicted pathogenicity were chosen for verification in mucosal melanoma samples cohort (Table S2).

NGS-based molecular tests were performed on 75 cases. Twenty-six cases were from prior studies [11,13]. Single nucleotide variants (SNV) and small insertion/deletions (indel) in genomic DNA were detected using Anchored Multiplex Polymerase chain reaction (PCR) by NGS (Table S3) [17]. A sequencing library targeting hotspots and exons in 99 cancer genes was generated using two hemi-nested PCRs. Using BWA-MEM Illumina MiSeq, 2 × 151 base paired-end sequencing results were aligned to the hg19 human genome reference [14]. For indel variant and SNV detection, a laboratory-developed insertion/deletion analysis algorithm and MuTech were used, respectively [18].

### 2.3. Statistical Analysis

The statistical associations between mutation of *NRAS*, *BRAF*, *KIT*, *SF3B1*, *IGF2R* and clinicopathologic features (patient’s age, stage, ulceration, mitotic index, lymphovascular invasion, perineural invasion, progression, recurrence, metastasis and death) were evaluated by Fisher’s exact tests. The number of months from diagnosis to development of locally recurrent or metastatic disease in the lymph nodes or distant organs was defined as progression-free survival (PFS). In patients with disease progression, time of death was equated to melanoma-related death. The number of months from initial diagnosis to patient’s death by any cause and related to melanoma were defined as overall survival (OS) and melanoma-specific survival (MSS), respectively. Kaplan–Meier plots and log-rank tests were done to visually assess the differences in OS, MSS and PFS between subgroups. Univariate analyses were performed with the Cox proportional hazards model. All covariates with *p* < 0.05 were included in the multivariate Cox proportional hazard model. All statistical analyses were done using the R statistical package [19]. A two-tailed *p* of less than or equal to 0.05 was considered to be statistically significant.

## 3. Results

**Figure 1:**
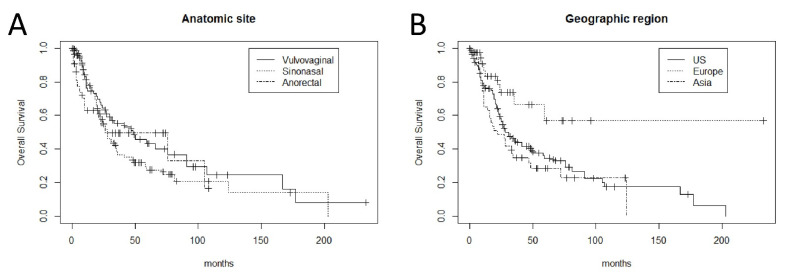
(**A**) Kaplan–Meier curves demonstrate no significant correlation between overall survival and anatomic sites (log-rank *p* = 0.3); (**B**) Kaplan–Meier curves demonstrate a significant correlation between overall survival and geographic areas (log-rank *p* = 0.01).

The study included 214 patients. The age of the patients ranged from 20 to 91 years (median, 65 years). The follow-up (FU) for all patients ranged from 0 to 233 months (median, 21 months). Progression (local recurrence and/or metastasis) developed in 156/214 (73%) patients. Metastases developed in 121/207 (58%) patients, with distant metastases seen in 88/207 (43%), with lung and/or liver being the most common metastatic sites. Death was documented in 117/214 (55%) patients. The patients were categorized into stage I/II versus stage III/IV to reflect whether metastasis was documented at time of diagnosis, due to incomplete data such as tumor size and tumor thickness in some cases, such as sinonasal melanomas. There were 168 patients with stage I/II, 45 with stage III/IV and 1 without known stage. There were no survival differences among the patients with vulvovaginal, sinonasal and anorectal melanomas (Figure 1A). Patients from Europe (42 patients; median FU, 11 months) have better OS (*p* = 0.01) in comparison to those from North America (124 patients; median FU, 24 months) and Asia (44 patients; median FU, 20 months) (Figure 1B).

Ulceration, lymphovascular invasion and perineural invasion were noted in 144/202 (71%), 41/209 (20%) and 26/209 (12%) cases, respectively. The number of mitoses identified per millimeter squared ranged from 0 to 100 (median, 7). Except for the correlation between *NRAS* and *IGF2R* mutation and presence of ulceration (*p* = 0.031) and lymphovascular invasion (*p* = 0.03), respectively, there were no associations between *NRAS*, *BRAF*, *KIT*, *SF3B1* and *IGF2R* mutations and clinicopathologic features.

**Figure 2:**
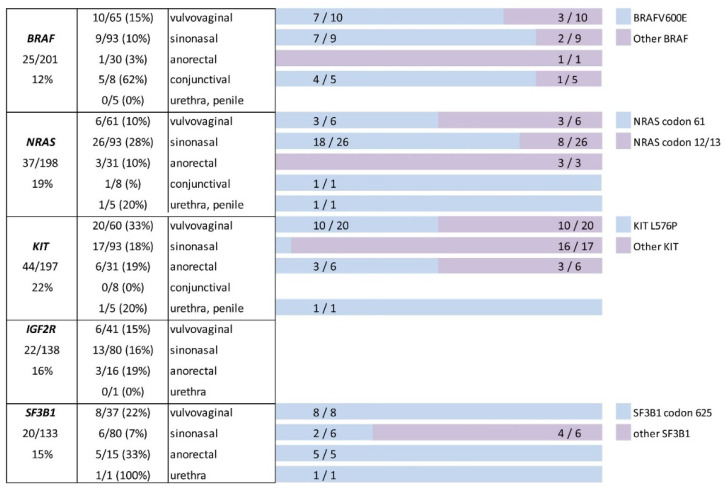
Summary of the molecular analyses. BRAF V600E, NRAS codon 61, KIT L576P and SF3B1 codon 625 are the most frequently detected mutations.

*NRAS*, *KIT*, *BRAF*, *IGF2R* and *SF3B1* mutational status were known in 198, 197, 201, 138 and 133 cases, respectively. *NRAS*, *KIT*, *BRAF*, *IGF2R* and *SF3B1* mutations were detected in 19% (37/198), 22% (44/197), 12% (25/201), 16% (22/138) and 15% (20/133) cases, respectively (Figure 2). Mutations of codons 61 and 12/13 of *NRAS* were detected in 62% and 38% of mutated cases, respectively. KIT L576P mutation was seen in 33%. BRAF V600E mutation was present in 72% of mutated cases. Mutations involving codon 625 of SF3B1 was detected in 80% of mutated cases (Figure 2).

**Figure 3:**
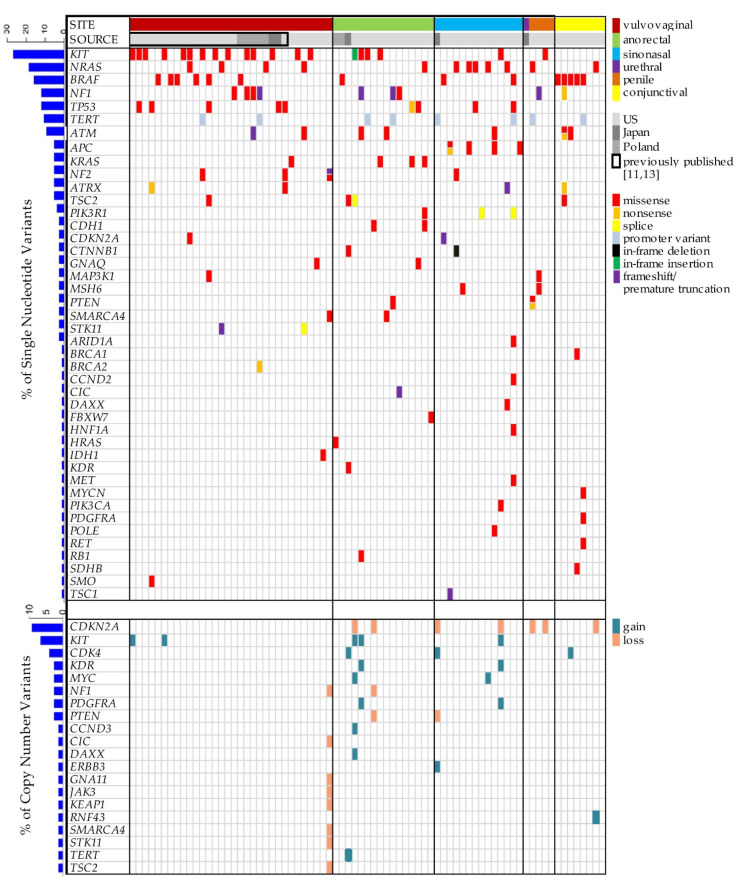
Summary of next-generation sequencing performed on 75 cases.

The NGS results of 49 mucosal melanomas (6 vulvar, 17 anorectal, 13 sinonasal, 8 conjunctival, 1 urethral and 4 penile), together with the published results of 26 vulvovaginal melanomas, are summarized in Figure 3 [11,13]. *BRAF*, *KIT* and *NRAS* were the most common recurrent mutations, seen more frequently in vulvovaginal and sinonasal melanomas in comparison to anorectal melanomas. In addition, mutations affecting the *APC*, *ATM*, *ATRX*, *CDH1*, *KRAS*, *NF1*, *NF2*, *PIK3R1*, *TSC2*, *TP53* and *TERT* promoter regions were noted. Of the triple (*BRAF*, *NRAS*, *NF1*)-negative cases, *APC*, *KIT* and *KRAS* were detected mainly in sinonasal, vulvovaginal and anorectal melanomas, respectively. Copy number variants, including loss of *CDKN2A* and gain of *KIT*, *CDK4* and *MYC*, were frequently seen in mucosal melanomas.

**Figure 4:**
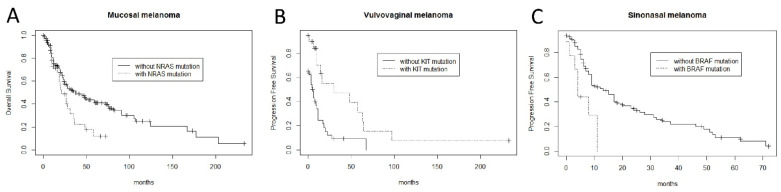
Kaplan–Meier curves demonstrate significant correlation between: (**A**) worse overall survival and *NRAS* mutation in mucosal melanomas (log-rank *p* = 0.028); (**B**) better progression-free survival and *KIT* mutation in vulvovaginal melanomas (log-rank *p* = 0.0021); (**C**) worse progression-free survival and *BRAF* mutation in sinonasal melanomas (log-rank *p* = 0.0045).

Univariate analyses are performed for the following variables: *NRAS*, *KIT*, *BRAF*, *SF3B1* and *IGF2R* mutation; stage; age; ulceration; mitoses; perineural invasion; lymphovascular invasion; and adjuvant therapy. Advanced stage (3 or 4, *p* = 0.018), age older than 65 years (*p* = 0.036) and presence of ulceration (*p* = 0.028) were significantly correlated with worse OS, respectively (Table S4). Whether the patient had received adjuvant therapy affected only PFS but not OS or MSS (Table S4). No significant correlation was observed between adjuvant therapy and overall survival. *NRAS* mutation significantly correlated with worse OS (*p* = 0.026) (Figure 4A) and worse MSS (*p* = 0.031) for all cases of mucosal melanomas. When stratified into subgroups, *KIT* mutation significantly correlated with improved PFS (*p* = 0.0021) for vulvovaginal melanomas (Figure 4B) and *BRAF* mutation with worse PFS for sinonasal melanomas (*p* = 0.0045) (Figure 4C and Table S4). No significant correlation with survival was seen for *IGF2R* and *SF3B1* mutations for all cases as well as for individual subgroups.

In multivariate analyses, *NRAS* mutation remained as an independent predictor of worse OS (*p* = 0.036) and worse MSS (*p* = 0.024). Higher stage (3 or 4) at diagnosis remained as independent predictor of worse OS as well as MSS (*p* = 0.026 and 0.0012, respectively) (Table 2).

The percentages of *NRAS*, *BRAF*, *KIT*, *IGF2R* and *SF3B1* mutations in different geographic regions are summarized in Table S5. *BRAF* mutation was more frequent in cases from Europe and Asia (*p* = 0.0066). *NRAS* mutation was detected more frequent in cases from North America versus Asia (*p* = 0.035).

## 4. Discussion

Mucosal melanomas are a rare and aggressive disease associated with frequent recurrence and distant metastases. The poor prognosis is likely a result of delay in diagnosis due to anatomic location. Overall survival (OS) has been cited to be highest in the vulvovaginal melanoma group, followed by sinonasal melanoma, and then anorectal melanoma [4,8,20]. Similar to findings reported in a series of 706 mucosal melanomas by Cui et al. [6], no significant correlation between survival and anatomic sites is observed in our study. Male gender, older age, depth of tumor, presence of ulceration and advanced stage are reported unfavorable prognostic variables [4,6,8,20,21,22,23]. In a series of 444 mucosal melanomas from a European population head and neck location, male gender, advanced tumor stage, nodal disease and incomplete resection status were independent risk factors for disease progression [8]. Older age and advanced stage are worse prognostic parameters in a German series of 161 patients [4]. In an analysis of 644 patients with vulvar melanoma, age less than 68 was an independent predictor of improved OS [24]. On the contrary, in some prior studies, age had no prognostic significance [6,25,26,27]. In our study, only advanced stage at diagnosis remains an independent negative prognosticator in multivariate analyses.

Geographic region might have a role in survival differences, and further studies are needed. We observe different frequencies of *BRAF* and *NRAS* mutations in cases from North America and Europe than from Asia. Similar differences in distribution of genetic variants between Western countries and Asia have been also reported in cutaneous melanomas [28]. Patients from Europe have significantly better OS (*p* = 0.01) in comparison to those from North America and Asia in our study. The reason is not known and further investigation is needed.

The prognostic role of histologic features in mucosal melanomas remains uncertain. In a study of 706 patients with mucosal melanomas by Lian et al. [29], depth of tumor invasion, number of lymph node metastases and distant metastases were independent prognosticators for OS in multivariate analyses and were similar for different mucosal sites. However, thickness has not been shown to be a predictor of survival in an analysis of 1824 mucosal melanomas since it cannot be determined in majority of mucosal melanomas due to the fragmented nature of the specimens and tangential nature of the histologic sections [22].

Dermal mitotic rate (≥2/mm^2^) has been shown to be an unfavorable prognosticator in vulvar and vulvovaginal melanomas [27,30]. On the contrary, mitotic rate had no significant impact on survival in a study of 86 mucosal melanoma patients by Cinotti et al. [23] and in another study of 85 cases by Tcheung et al. [31]. Similarly, we do not observe increased mitotic activity to be significantly associated with reduced survival in mucosal melanoma patients in our series.

The presence of ulceration correlates with worse OS in univariate analyses in our series. Similarly, Heppt et al. [8] showed that presence of ulceration is an important predictor of shorter OS. On the contrary, ulceration has no prognostic significance for OS in a series of 706 prospectively-followed patients with mucosal melanoma [6]. There is a trend toward significance for worse PFS and lymphovascular invasion in our study. Keller et al. [32] observed that lymphovascular invasion was strongly correlated with decreased survival in mucosal melanoma patients. In a series of 46 patients with anorectal melanoma, the presence of perineural invasion was identified as an independent predictor of disease-specific mortality in multivariate analysis [33].

The Mitogen-Activated Protein Kinase (MAPK) pathway plays an important role in melanoma pathogenesis. V-raf murine sarcoma viral oncogene homolog B (*BRAF*) and neuroblastoma RAS viral oncogene homolog (*NRAS*) mutations are different in mucosal melanomas in comparison to cutaneous melanomas [34]. *NRAS* mutant melanomas (NEMOs) are reported to be associated with increased risk of visceral and central nervous system metastases in comparison to wild-type cutaneous tumors [35]. The presence of *NRAS* mutations correlated with worse OS in a series of 2793 cutaneous melanomas by Bai and colleagues [36]. Similarly, we observe that *NRAS* mutation correlated with worse OS and MSS in mucosal melanomas. Although mutations at codon 61 are seen in both cutaneous and mucosal melanomas, mutations involving codons 12 and 13 (G13D, G12A and G12D) occur more frequently in mucosal melanomas [34]. Dumaz et al. [34] reported *NRAS* mutations in 12% (179/1454) of mucosal melanomas with 54% (96/179) and 46% (83/179) located on Q61 and G12/G13, respectively. Similarly, we observe *NRAS* mutations in 19% (37/198) with 62% (23/37) on codon Q61 and 38% (14/37) on codon G12/13.

Melanomas that harbor *NRAS* mutation, either previously untreated and those progressed on immunotherapy, might be targeted by MEK inhibitor such as Binimetinib or Pimasertib [37,38]. In melanoma cell lines with activating *NRAS* mutations, combination of PI3K or AKT inhibitors with MEK inhibitors has demonstrated synergistic inhibition [39]. For patients with *NRAS* mutant melanomas, there have been several clinical trials of combination therapy: combination of CDK4/6 inhibitor (LEE011) and MEK inhibitor (MEK162) in phase Ib/II clinical trial [40], and combination of RAF inhibitor (LXH254) with ERK1/2 inhibitor (LTT462) or Trametinib (MEK inhibitor) in phase Ib clinical trial [41].

*BRAF* mutations have been reported in 8% (107/1339) of mucosal melanoma involving V600E in 63% (67/107) and another codon in the remaining 37% (40/107) [34]. In line with published results, we observe *BRAF* mutations in 12% (25/201) of studied mucosal melanomas with V600E and variants detected in 72% and 28%, respectively. In conventional melanoma, *BRAF*-mutated tumors have been reported to be more aggressive than the corresponding wild-type tumors [42]. Although no correlation with prognosis is observed for all mucosal melanomas in the current series, *BRAF* mutation correlates with worse PFS for sinonasal melanomas when subgroups are analyzed. V600E mutation is observed in mucosal melanomas involving half of the conjunctival melanomas in our study [43]. D594G, G469A and K601E are the frequently observed BRAF variants in mucosal melanomas [34]. Similarly, A581S, L579Q, G469R, D594G and G466V BRAF variants are detected in our series. BRAF inhibitors have been shown to significantly lengthen PFS and OS in patients with melanoma harboring BRAF V600 mutations (V600E and V600K) [42,44]. However, BRAF inhibitors target tumors harboring BRAF V600E and not *BRAF* variants; therefore, other treatment modalities such as RAF inhibitor are currently under study [42,43,44,45].

Alterations in *KIT* play an important role in tumor growth, proliferation and metastases in a variety of cancer [46]. *KIT* mutations in primary melanoma are composed of missense substitutions on different exon distribution in comparison to *KIT*-mutated gastrointestinal stromal tumor [47]. *KIT* mutation has been reported from 10% to 21% of mucosal melanomas [47,48]. The overall *KIT* alteration frequency of 19% to 39%, with the L576P mutation being the most common mutation and *KIT* amplification seen in 10–26% [47,48]. Patients whose tumors harbor *KIT* L576P and K642E mutations can be targeted with *KIT* inhibitors such as imatinib, sunitinib, dasatinib and nilotinib [42]. While *KIT* mutation and/or amplification were reported to be adverse prognostic marker in melanomas in the Asian population, *KIT* mutation correlates with better PFS for vulvar melanomas in our series [49,50]. It could be that a large percentage of acral melanoma was included in these published series.

*SF3B1* (splicing factor 3 subunit B1) mutation has been reported to be associated with good prognosis as well as late metastases in different series of uveal melanomas [51,52]. *SF3B1*, a mutation of codon 625 seen in 84% of the cases, does not correlate with prognosis in our series of mucosal melanomas. Recurrent R625C and R625H mutations are the main mutations reported in uveal, vulvovaginal and anorectal melanomas [2,51,52]. While Newell et al. [5] reported *SF3B1* mutations most frequently in mucosal melanomas from Europe, we observe no geographical differences for *SF3B1* in our study.

In a recent study by Iida et al. [10], the *IGF2R* variants were detected in 32% of cases, making it the most commonly mutated gene in mucosal melanoma. Our results do not confirm the high frequency of highly pathogenic *IGF2R* variants, with 18% of cases harbored the mutation. Although IGF2R L252V mutation is frequently detected in our study (data not shown), it is most likely benign polymorphism, reported in almost 14% of population worldwide [53]. Excluding the low and moderate pathogenic variants from our analysis and focusing only on highly pathogenic variants may be the reason of observed differences in *IGF2R* mutation frequency between ours and study by Iida et al. [10]. Although our results do not confirm the direct role of *IGF2R* mutations in driving mucosal melanoma development, there are data suggesting its more complex role. As shown in cutaneous melanoma, reduced expression of *IGF2R* inhibits the metastatic potential of melanoma cells [54]. Lately, it has been shown that the IGF axis with an emphasis on the *IGF2R* gene is responsible for metastatic niche formation by transforming the normal fibroblast into cancer-associated fibroblasts (CAFs) [55]. These results suggest that *IGF2R* variants may have a complex, yet so far unknown role in driving mucosal melanoma progression, for which an explanation requires further research.

Recent studies of mucosal melanomas by whole exome sequencing demonstrated that mucosal melanomas have a low mutational burden, with frequent structural variants commonly affecting *CDK4*, *MDM2* and *TERT* [5,9]. From the available NGS data of our cases, copy number variants affecting *CDKN2A* and *KIT* are frequently seen in mucosal melanomas. In addition to commonly noted *BRAF*, *NRAS* and *KIT*, mutations affecting the *APC*, *ATM*, *ATRX*, *CDH1*, *KRAS*, *NF1*, *NF2*, *PIK3R1*, *TSC2*, *TP53* and *TERT* promoter regions are noted. When the mucosal melanoma is triple (*BRAF*, *NRAS*, *NF1*)-negative, *KIT* is the most commonly mutated gene in vulvovaginal melanomas, while *APC* and *KRAS* are detected mainly in sinonasal and anorectal melanomas, respectively. Co-mutation of *KIT* and *NF1*, previously reported by Hintzsche et al. [56] and seen in 2 vulvar melanomas of our prior study [11], was detected in one anorectal melanoma in the current series. Although *SPRED1*, *HLA-A* and *CHD8* are not included in our NGS panel, well-known driver genes of melanomas such as *KRAS*, *NF1*, *SF3B1*, *TP53* and *TERT* are detected in our mucosal melanoma cases.

Our study has several limitations. Due to the multicenter nature of our study, the patients included in our study did not receive uniform surgical and/or medical treatment. A high failure rate was observed with next-generation sequencing tests performed on old archival materials. Nevertheless, our study includes a significant number of these rare subtypes of melanomas for analyses.

## 5. Conclusions

In conclusion, our series of mucosal melanomas confirms frequent mutation of melanoma driver genes, including *BRAF*, *NRAS*, *KIT*, *KRAS*, *SF3B1*, *NF1*, *TP53* and *TERT*. In multivariate analyses *NRAS* mutation remains a predictor of worse survival independent of stage in mucosal melanomas. *KIT* mutation correlates with improved PFS for vulvovaginal melanomas and *BRAF* mutation with worse PFS for sinonasal melanomas only in univariate analyses. The significance of frequently mutated *IGF2R* in mucosal melanomas remains unclear. There appears to be some geographical differences in molecular alterations; however, larger cohorts of mucosal melanomas are needed for further investigation.

## Figures and Tables

**Table 1:** Summary of cases analyzed by next generation sequencing (NGS) and Sanger sequencing.

Melanoma Subtype	*BRAF NRAS* *KIT* NGS	Sanger				
		*BRAF*	*NRAS*	*KIT*	*SF3B1*	*IGF2R*
Vulvovaginal	32	33	29	28	37	41
Sinonasal	13	80	80	80	80	80
Anorectal	17	13	14	14	15	16
Conjunctival	8	0	0	0	0	0
Penile	4	0	0	0	0	0
Urethra	1	0	0	0	1	1
Total	75	126	123	122	133	138

**Table 2:** Multivariate Cox proportional hazards models.

	Overall Survival		Melanoma-Specific Survival	
	Hazard Ratio	*p*-Value	Hazard Ratio	*p*-Value
*NRAS* mutation	1.71	0.036 *	1.80	0.024 *
Stage (3–4 versus 1–2)	1.71	0.026 *	2.11	0.0012 *
Age (> 65 years)	1.41	0.10	-	-
Ulceration	1.49	0.11	-	-

* *p* < 0.05, statistical significance.

## Data Availability

All data generated or analyzed during this study are included in this published article and its supplementary information files.

## Supplementary Materials

The following are available online at https://www.mdpi.com/article/10.3390/cells10092216/s1, Table S1: Primers for polymerase chain reaction amplification and sequencing. Table S2: IGF2R methods. Table S3: The single nucleotide variants and indel gene targets covered by the next-generation sequencing tests are as follows (exons). Table S4: Univariate Cox proportional hazards models. Table S5: Geographic distribution of *NRAS*, *BRAF*, *KIT*, *SF3B1* and *IGF2R* mutations.
