# Supplementary material for: Prognostic Roles of BRAF, KIT, NRAS, IGF2R and SF3B1 Mutations in Mucosal Melanomas
Source: Cells. 2021 Aug 27;10(9):2216. doi: 10.3390/cells10092216 (PMC8468625; doi:10.3390/cells10092216)
Supplement: Supplementary file 1 [file cells-10-02216-s001.zip › cells-1348032-supplementary.pdf]

cells MDPI

Supplemental Table S1: Primers for polymerase chain reaction amplification and sequencing.

Gene	Forward	Reverse
BRAF exon 15	AG-CATCTCAGGGCCAAAAAT	TGCTTGCTCTGA-TAGGAAAATG
KIT exon 11	CTCTCCAGAG-TGCTCTAATGAC	AGCCACTGGAG-TTCCTTAAAG
KIT exon 13	CGGCCATGACTGTCGCTG-TAA	CAATAAAAGGCAGCTT-GGACACG
KIT exon 17	CACAG-GAAACAATTTTTATCGAAAGTTGAAAC	TGAATTTAAATGGTTTTCTTTTCTCCTCCAAC
NRAS exon 1	GATGTGGCTCGCCAATTAAC	CCGACAAGTGAGAGA-CAGGA
NRAS exon 2	TTGCATTCCCTGTGGTTTTT	TGGTAAC-CTCATTTCCCCATA
SF3B1 codon 625 (exon 14)	CCAACTCATGACTGTCCTTTCTT	TGCCAGGACTTCTTGCTTTT
SF3B1 codon 666 (exon 14)	TTTGCTGTTGTAGCCTCTGC	CAACTTAC-CATGTTCAATGATTTC
SF3B1 codon 700 (exon 15)	TTGAGAGAATCTGGATGA-TATTGTG	GGCGGATACCCTTCCA-TAAA
IGF2R exon 2	TGTAAAACGACGGCCAG-Taactagagagaagtttaatttttga	CAGGAAACAGCTATGAC-Cgcaaaattggggaatcata
IGF2R exon 6	TGTAAAACGACGGCCAG-Tgtctaagggtacgtgtgatt	CAGGAAACAGC-TATGACCcaggtccttgctggagat
IGF2R exon 8	TGTAAAACGACGGCCAGTt-gcattaagctgcatgaaaca	CAGGAAACAGC-TATGACCcctctctcgatccccttctt
IGF2R exon 16	TGTAAAACGACGGCCAG-Tcagccttggagtgcttctg	CAGGAAACAGC-TATGACCcccaccacaggcatgag-tat
IGF2R exon 43	TGTAAAACGACGGCCAGTg-cagtcttcccttatgtctgg	CAGGAAACAGC-TATGACCcggcattgctggtttaattt
IGF2R exon 46	TGTAAAACGACGGCCAG-Tctgtggcagcaggaccac	CAGGAAACAGC-TATGACCcaaaactgaccccaa-gattagc

Supplemental Table S2: IGF2R methods. In order to select promising variants for further experimental validation, we performed the hard filtering according to GATK good practices with the criteria as follows:

For the single nucleotide variants:
Variant Confidence/Quality by Depth < 2.0
Phred-scaled p-value using Fisher's exact test to detect strand bias > 60;

RMS Mapping Quality < 40;
Z-score From Wilcoxon rank sum test of Alt vs. Ref read mapping qualities < -12.5;
Z-score from Wilcoxon rank sum test of Alt vs. Ref read position bias < -8.0.

For the indels:
Variant Confidence/Quality by Depth < 2.0;
Phred-scaled p-value using Fisher's exact test to detect strand bias > 200;
Z-score from Wilcoxon rank sum test of Alt vs. Ref read position bias < -20.

Afterward, we selected only the variants characterized by QUAL >= 30 (Phred-scaled quality score) and DP >= 5 (read depth at particular position). Finally, we focused only on variants present in coding sequences of particular genes. Only detected variants with a high score for predicted pathogenicity were chosen for verification in the mucosal melanoma sample cohort, as shown in the table below.

**Primary site**	**IGF2R variants**
Vulvovaginal	c.4855A>G|p.Arg1619Gly; c.6059A>G|p.Asn2020Ser;<br>c.7376C>T|p.Ala2459Val<br>c.4687G>A|p.Val1563Met<br>c.2609T>A|p.Val870Glu
Sinonasal	c.259G>A|p.Asp87Asn<br>c.685C>T|p.Pro229Ser<br>c.754C>G|p.Leu252Val<br>c.6329T>C|p.Ile2110Thr<br>c.2156G>C|p.Arg719Thr<br>c.923A>G|p.Tyr308Cys<br>c.910T>C|p.Ser304Pro<br>c.6913G>C|p.Ala2305Pro
Anorectal	c.6833G>T|p.Cys2278Phe

Supplemental Table S3: The single nucleotide variants and indel gene targets covered by the next-generation sequencing tests are as follows (exons).

ABL1(4–7), AKT1 (3,6), ALK (21–23,25), APC (16), ARID1A (1–20), ATM (1–63), ATRX(1–35), AURKA (2,5–8), BRAF (11,15), BRCA1 (2–23), BRCA2 (2–27), CCNB1(2,[3-partial],5,[6-partial],7), CCND2 ([2-partial],3-4,[5-partial]), CCND3 (2–5-partial), CCNE1 (3–8,10,12), CDH1 (1–16), CDK4 (2–7), CDK6 (6), CDKN2A (1–3), CIC (1–20), CSF1R (7,22), CTNNB1 (3), DAXX (1–8), DDR2 (12–18), DDX3X (1–17), EGFR (3,7,15,18–21), ERBB2 (8,10,19–21,24), ERBB3 (2–3,7–8), ERBB4 (3–4,6–9,15,23), ESR1 (8), EZH2 (16), FBXW7 (1–11), FGFR1 (4,7–8,13,15,17), FGFR2 (7,9,12,14), FGFR3 (7–9,14–6,18), FLT3 (11,14,16,20), FOXL2 (1), GNA11 (5), GNAQ (4–5), GNAS (6–9), H3F3A (2), HNF1A (3–4), HRAS (2–3), IDH1 (3–4), IDH2 (4), JAK2 (11,13–14,16,19), JAK3 (4,13,16), KDR (6, 7,11,19,21,26–27,30), KEAP1 (2–6), KIT (2,8–11,13–15,17–18), KRAS (2–5), MAP2K1 (2,3,6–7), MAP3K1 (1–20), MDM2 (2–4,6,8,10), MDM4 ([4-partial],5-6,[7,9–11-partial]), MEN1 (2–10), MET (2,11,14,16,19,21), MITF (1–partial), MLH1 (12), MPL (10), MSH6 (1–10), MSI, MYC (1–3), MYCN (3), NF1 (1–58), NF2 (1–5), NKX2-1 (1-partial), NOTCH1 (25–27,34), NPM1 (11), NRAS (2–5), PDGFRA (12,14–15,18,23), PIK3CA (2,5,7–8,10,14,19,21), PIK3R1 (1–10), POLE (9–14), PTCH1 (1–23), PTEN (1–9), PTPN11 (3,13), RB1 (1–27), RET (10–11,13–16), RHOA (2–3), RNF43 (2–10), ROS1 (36–38), SDHB (1–8), SMAD2 (7), SMAD4 (2–12), SMARCA4 (3–36), SMARCB1 (2,4,5,9), SMO (3,5–6,9,11), SRC (14), STAG2 (3–

34), STK11 (1–9), SUFU (1–12), TERT (1), TP53 (1–11), TP63 (1–14), TSC1 (3–23), TSC2 (2–42), TSHR (10), VHL (1–3).

Supplemental Table S4: Univariate Cox proportional hazards models.

		**Overall survival**		**Melanoma-specific survival**		**Progression-free survival**	
		**Hazard ratio**	***p* value**	**Hazard ratio**	***p* value**	**Hazard ratio**	***p* value**
***NRAS* mutation**	198	1.70	0.028 *	1.75	0.03*	1.27	0.25
Vulvovaginal	61	2.93	0.032 *	2.74	0.07	1.39	0.36
Sinonasal	93	1.4	0.27	1.55	0.19	1.21	0.58
***KIT* mutation**	197	0.98	0.91	0.89	0.66	0.79	0.24
Vulvovaginal	60	0.72	0.36	0.59	0.19	0.34	0.0021*
***BRAF* mutation**	201	0.90	0.76	0.90	0.76	0.97	0.91
Sinonasal	94	1.33	0.54	1.31	0.61	3.06	0.0045*
***SF3B1* mutation**	133	0.66	0.27	0.60	0.23	0.59	0.12
***IGF2R* mutation**	138	0.58	0.085	0.62	0.17	0.80	0.41
Stage (3-4 versus 1-2)	213	1.69	0.016 *	2.11	0.0009*	346	<0.001*
Age (> 65 years)	214	1.45	0.048 *	1.24	0.29	0.84	0.28
Ulceration	202	1.65	0.027 *	1.49	0.098	1.08	0.68
Mitoses (≥2)	199	0.94	0.78	0.99	0.97	1.33	0.17
Perineural invasion	209	0.88	0.68	0.87	0.68	1.48	0.099
Lymphovascular invasion	209	0.93	0.78	1.08	0.76	1.53	0.031*
Adjuvant therapy	198	0.96	0.857	1.11	0.63	1.63	0.0052*

* $p < 0.05$, statistical significance

Supplemental Table S5: Geographic distribution of *NRAS*, *BRAF*, *KIT*, *SF3B1* and *IGF2R* mutations.

Gene	North America	Europe	Asia	North America versus Europe *p* value	North America versus Asia *p* value	Europe versus Asia *p* value
NRAS	25/114 (22%)	9/41 (22%)	3/43 (7%)	1.0	0.035*	0.65
BRAF	15/117 (13%)	9/41 (22%)	1/43 (2%)	0.21	0.071	0.0066*
KIT	30/119 (25%)	7/35 (20%)	7/43 (16%)	0.65	0.29	0.77
SF3B1	9/64 (14%)	3/29 (10%)	8/40 (20%)	0.75	0.43	0.34
IGF2R	13/66 (20%)	3/31 (10%)	9/41 (22%)	0.26	0.81	0.21

* $p < 0.05$, statistical significance
